# Cystic echinococcosis in Poland: genetic variability and the first record of *Echinococcus granulosus* sensu stricto (G1 genotype) in the country

**DOI:** 10.1007/s00436-017-5618-4

**Published:** 2017-10-03

**Authors:** Rusłan Sałamatin, Jerzy Kowal, Paweł Nosal, Sławomir Kornaś, Danuta Cielecka, Dawid Jańczak, Waldemar Patkowski, Jakub Gawor, Vadim Kornyushin, Elzbieta Golab, Viliam Šnábel

**Affiliations:** 1 Department of General Biology and Parasitology, Medical University of Warsaw, Chałubińskiego 5, 02-004 Warsaw, Poland; 2 Department of Parasitology, National Institute of Public Health – National Institute of Hygiene, Chocimska 24, 00-791 Warsaw, Poland; 3 Department of Zoology and Ecology, Faculty of Animal Sciences, University of Agriculture in Krakow, al. Mickiewicza 24/28, 30-059 Krakow, Poland; 4 Department of General, Transplant and Liver Surgery, Medical University of Warsaw, Banacha 1a, 02-097 Warsaw, Poland; 5 W. Stefański Institute of Parasitology, Polish Academy of Sciences, Twarda 51/55, 00-818 Warsaw, Poland; 6 I. I. Schmalhausen Institute of Zoology, National Academy of Sciences of Ukraine, Khmelnytskoho 15, Kyiv, 01601 Ukraine; 7 Institute of Parasitology, Slovak Academy of Sciences, Hlinkova 3, 04001 Košice, Slovakia

**Keywords:** *Echinococcus granulosus*, Poland, DNA sequencing, Genotype, Sheep, Human, Pig

## Abstract

Cystic echinococcosis is one of the most important zoonotic diseases affecting humans and livestock worldwide, and is endemic in Poland. A set of six isolates on larval stages of *Echinococcus granulosus* sensu lato tapeworms collected from three humans, two pigs and one sheep from Polish foci of CE was examined by DNA sequencing of two mitochondrial genes (*cox1*, *rrnS*). The results demonstrated the presence of *E. canadensis* and *E. granulosus* sensu stricto in the investigated hydatid cysts. The former species was found in all five isolates from pigs and humans derived from central Poland. In a sheep hydatid cyst originating from Lesser Poland Voivodeship in southern Poland, *E. granulosus* s. s. (G1 genotype) was identified. This is the first report of an unambiguously autochthonous infection with *E. granulosus* s. s. in Poland. The global distribution and host affiliations of the commonly occurring G1 microvariant with nucleotide change 56C/T in *cox1*, detected here in Polish sheep, are discussed. The finding that sheep harboured *E. granulosus* s. s. may have important consequences for developing effective hydatid control programmes in Poland due to its longer maturation rate in dogs compared with *E. canadensis* G7. This may lead to greater expenditures for purchasing anthelmintics to provide an appropriate dosing regime in sheep-raising areas of the country.

## Introduction

The larval stages of the tapeworm *Echinococcus granulosus* sensu lato are the causative agent of cystic echinococcosis (CE), one of the most important cestode infections causing morbidity and mortality in humans and significant economic losses in livestock. Around one million or more people are currently suffering from CE globally, and the financial burden of the disease on the livestock industry is substantial, with up to $2 billion lost annually (Torgerson and Macpherson 2011).

According to the current nomenclature, *E. granulosus* s. l. circulating in Europe has been subdivided into *E. granulosus* sensu stricto (“sheep strain” and “buffalo strain”, genotypes G1 and G3), *Echinococcus equinus* (“horse strain”, G4), *Echinococcus ortleppi* (“cattle strain”, G5) and *Echinococcus canadensis* (“camel strain”, G6; “pig strain”, G7; “two cervid strains”, G8 and G10) (Romig et al. 2015). Human cystic echinococcosis is caused predominantly (approximately 90% of cases worldwide) by *E. granulosus* s. s., which has the most cosmopolitan distribution and is largely transmitted in areas with extensive sheep farming (Alvarez Rojas et al. 2014). That species is followed by *E. canadensis* (genotypes G6–G10), globally responsible for about 10% of human infections. Among the remaining species traditionally classified as *E. granulosus* sensu lato, only nine human infections with *E. ortleppi* (G5) and no infection with *E. equinus* (G4) have been reported to date (Alvarez Rojas et al. 2014; Grenouillet et al. 2014).

In Poland, in 2015, over 4500 cases of cystic echinococcosis in farm animals were recorded according to the report of the European Food Safety Authority (EFSA and ECDC 2016), and in 99.56% of those cases, pigs were the intermediate hosts. Only 0.43 and 0.01% of cases of cystic echinococcosis were recorded in sheep and cattle, respectively. All genotyped metacestodes originating from humans, domestic pigs and the European beaver belonged to the G7 genotype of *E. canadensis* (summarized in Cardona and Carmena 2013; Alvarez Rojas et al. 2014). In geographical terms, transmission of the G7 genotype is largely confined to a contiguous zone in central and eastern Europe including the Baltic region. No records about the circulation of highly pathogenic *E. granulosus* s. s. in Poland and/or neighboring countries are available so far.

The study was conducted to extend the knowledge about the genotype spectrum of *Echinococcus granulosus* tapeworms circulating in sheep, pigs and humans in Poland.

## Material and methods

### Sample collection

*Echinococcus* protoscoleces were collected from the livers of naturally infected pigs (two isolates from the Masovian Voivodeship in central Poland) and sheep (one isolate from Podhale district in Lesser Poland Voivodeship in southern Poland). Three human samples were derived from surgically removed hydatid cysts from patient livers at the Department of General Transplant and Liver Surgery, Medical University of Warsaw (central Poland). Sheep and pig samples were frozen at −20 °C, subsequently defrosted and stored in 70% ethanol. Human samples were stored in 70% ethanol.

### DNA extraction

Fragments of ethanol-preserved hydatid cyst samples were dried at room temperature, homogenised and subjected to DNA isolation by the silica-guanidinium procedure (Boom et al. 1999).

### DNA amplification and sequencing

A gene fragment of cytochrome *c* oxidase subunit 1 (*cox1*, 396 bp) was amplified with JB3/JB4.5 primers (Bowles et al. 1993) from mitochondrial DNA of all isolates. In the sheep isolate, a portion of the small subunit ribosomal RNA gene (*rrnS*, 372 bp) was amplified with P60/P375 primers (Dinkel et al. 1998). Amplified PCR products of both mitochondrial genes were then subjected to automated Sanger sequencing.

### Sequence analysis

The sequences of the *cox1* gene were compared to the reference sequences (Bowles et al. 1992) of *E. granulosus* (genotypes G1–G3), *E. equinus* (genotype G4), *E. ortleppi* (genotype G5) and *E. canadensis* (genotypes G6 and G7). The sequences of the mitochondrial small subunit rRNA were compared to the reference sequences (Dinkel et al. 2004; Busi et al. 2007) of *E. granulosus* s. s. (G1 and G3) and *E. canadensis* (G6 and G7). The multiple sequence alignments were performed using the CLC Main Workbench 7 software. Generated haplotypes were identified through BLASTn analysis. To distinguish synonymous and non-synonymous mutations, EMBOSS transeq software for deriving protein sequences was used. The sequences reported in this paper were deposited in the GenBank database with the accession numbers KJ831062, KM191134, MF580386 and MF580387.

### Morphological analysis

Protoscoleces were mounted in Hoyer’s medium (Cielecka et al. 2009) and pressure was applied to the coverslip to cause the hooks to lie flat. All measurements were taken by the same person (D. C.) using a calibrated eyepiece micrometer under oil immersion. The number of rostellar hooks, the length of the blades of large and small hooks and total length of the large and the small hooks were considered. The hooks were measured according to Ponce Gordo and Cuesta Bandera (1997). Only invaginated, viable protoscoleces were analysed.

## Results

Based on the sequences the *cox1* gene fragment five isolates (2 from pigs and 3 from humans) were classified as bearing the G7 genotype. The isolate from sheep was identified as the G1 genotype based on the sequence of the fragments of *cox1* and *rrn*S genes and herein was provisionally designated as G1A microvariant sensu Šnábel et al. (2009) (Table 1).

**Table 1:** Previous available records of G1A microvariant of *Echinococcus granulosus* sensu stricto

Region/countries	Host (*n*)	GenBank accession numbers	References
Africa			
Algeria	Human (2)	KR349028	Zait et al. (2016)
Ethiopia	Sheep (3), cattle (1), camel (1)	AB650531	Hailemariam et al. (2012)
Morocco	Camel	EF367279	
Morocco	Cattle (2)	EF367280, EF367283	
Morocco	Goat	EF367281	
Morocco	Mule	EF367285	
Morocco	Sheep (2)	EF367282, EF367284	
Tunisia	Cattle (3), human (3), sheep (1)		M’rad et al. (2005)
Tunisia	Cattle (6), human (2), sheep (1)		M’rad et al. (2010)
Tunisia	Donkey (7), sheep (4), cattle (1)	KM014642	Boufana et al. (2014)
Tunisia	Human	KM014643	Boufana et al. (2014)
Tunisia	Sheep (2), wild boar (1)	KM014641	Boufana et al. (2014)
Africa (country of origin not known	Red-tailed guenon	JX068640	Boufana et al. (2012)
Asia			
Armenia	Cattle (6)	KX020338, KX020339, KX020344, KX020345, KX020368,KX020372	
Armenia	Goat	KX020377	
Armenia	Human (5)	KX020337, KX020341, KX020359, KX020365, KX020367	
Armenia	Sheep (8)	KX020336, KX020357, KX020383, KX020386, KX020388, KX020391, KX020392, KX020402	
China, Qinghai province	Sheep (3)	AB491421	Nakao et al. (2010)
China, Xinjiang province	Human (2)	AB491439, AB491447	Nakao et al. (2010)
China, Xinjiang province	Human (3), dog (11)	DQ356877	Bart et al. (2006)
Kazakhstan	Dog	KT001396	Boufana et al. (2015)
Mongolia	Human	AB893246	Ito et al. (2014)
Mongolia	Human (2)	AB787546, AB787548	
Mongolia	Sheep (2)	AB787531, AB787538	
Russia (Altai Krai)	Human	AB688139	Konyaev et al. (2012a)
Russia (Novosibirsk Oblast)	Human	AB688140	Konyaev et al. (2012a)
Europe			
Albania	Sheep	KU925433	Kinkar et al. (2016)
Austria	Human	AJ508019	Obwaller et al. (2004)
Bulgaria	Human	KY235681	
Greece	Sheep	KM245580	
Hungary	Human	JF690976	Šnábel et al. (2016)
Italy	Sheep (3)		Busi et al. (2007)
Moldova	Sheep (6), cattle (2)	KJ782437	Umhang et al. (2014)
Poland	Sheep	KJ831062	this study
Portugal	Sheep	HF947559	Beato et al. (2013)
Romania	Cattle	KU925431	Kinkar et al. (2016)
Russia (Permskiy Krai)	Sheep	AB777906	Konyaev et al. (2013)
Spain	Sheep	KU925419	Kinkar et al. (2016)
Middle East			
Iran	Camel	JQ250814	Yanagida et al. (2012)
Iran	Camel	HM563013	
Iran	Dog	KP339046	Gholami et al. (2016)
Iran	Dog	JN604098	Parsa et al. (2012)
Iran	Goat	KR337820	
Iran	Sheep (11), cattle (7), human (6)	KP859560	Farhadi et al. (2015)
Iran	Human	AB677811	Pezeshki et al. (2013)
Iran	Human (2)	JQ250810, JQ250812	Yanagida et al. (2012)
Iran	Sheep	JQ219962	
Iran	Sheep	KP751431	
Iran	Sheep	HM563012	
Iran	Human	KM513627	Sharbatkhori et al. (2016)
Iran	Sheep	KT074944	Sharbatkhori et al. (2016)
Iran	Cattle	KT074945	Sharbatkhori et al. (2016)
Iran	Camel	KT074946	Sharbatkhori et al. (2016)
Iran	Sheep (3)	JQ250809, JQ250811, JQ250813	Yanagida et al. (2012)
Iran	Water buffalo (6)	HM130586-HM130591	Pour et al. (2011)
Iran	Cattle (2)	KT254113, KT254124	
Jordan	Sheep (2)	AB688599, AB688600	Yanagida et al. (2012)
Palestine	Sheep (2)	KC109657, KC109659	Adwan et al. (2013)
Turkey	Cattle (14)	KU925358, KU925364, KU925370, KU925372, KU925373, KU925376, KU925378, KU925379, KU925384, KU925385 KU925386, KU925409-KU925411	Kinkar et al. (2016)
Turkey	Sheep (8)	KU925385, KU925391, KU925392, KU925401, KU925402, KU925404, KU925405, KU925412	Kinkar et al. (2016)
Turkey	Human (2)	HQ717148	Eryıldız and Şakru (2012)
Turkey	Sheep	AJ508012	Obwaller et al. (2004)
Turkey	Sheep	KM100575	
Turkey	Cattle	EF689726	Utuk et al. (2008)
Turkey	Cattle (2)	EU178104	Vural et al. (2008)
Turkey	Human (2)	JF775379	Šnábel et al. (2009)
Turkey	Sheep	JF775380	Šnábel et al. (2009)
Turkey	Sheep	JN810793	
Turkey	Water buffalo (2)	HM598457, HM598459	Beyhan and Umur (2011)
South America			
Argentina	Cattle	KX039951	Laurimäe et al. (2016)
Brazil	Sheep	HF947571	Beato et al. (2013)

*n* number of host specimens

### cox1

The sequence of the *cox1* gene of the isolate from sheep had the highest level of similarity to reference genotypes G1–G3 of *E. granulosus* s. s., with one substitution (56C/T) compared to the reference G1 genotype, two substitutions (66T/C, 257C/T) compared to previously assigned G2 genotype, and three substitutions (56C/T, 66C/T, 257C/T) compared to the G3 genotype. The non-synonymous nucleotide change with a thymine at position 56, which induces substitution of alanine with valine, is typical of the G2 genotype, but the remaining nucleotides of sheep isolate corresponded to the sequence pattern of the G1 genotype.

**Fig. 1:**
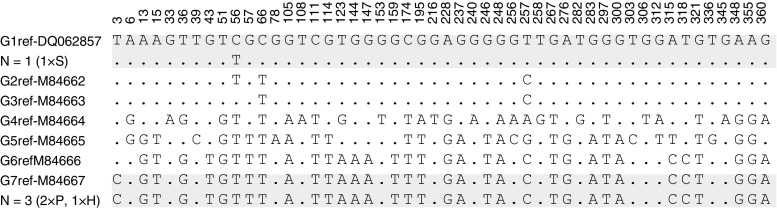
Alignment of variable sites in the partial *cox1* sequences (366 bp) with available sequences of related reference genotypes. N number of isolates detected for each variant, S sheep host, P pig host, H human host

The *cox1* sequences obtained from human and swine isolates were identical to the reference sequence for genotype G7 of *E. canadensis*. Multiple sequence alignments are presented in Fig. 1.

### *rrnS*

The sequence of the *rrnS* gene obtained from the sheep isolate had 100% homology with a G1 reference sequence, thus corroborating genotypic structure characteristic for *E. granulosus* s. s. G1.

### Morphological characteristics of protoscoleces

The rostellar hook characteristics of protoscoleces of Polish sheep origin are shown in Table 2. Morphometrical data suggested that rostellar hooks in the examined sheep were apparently shorter than those previously measured from pig and humans in the same region of Europe (Poland and Ukraine), whereas a high similarity in hook sizes was found in relation to previously analysed sheep isolates from UK and Spain.

**Table 2:** Morphometrical data of protoscoleces of *Echinoroccus granulosus* sensu lato from pig and sheep in Europe

Reference	Geographical origin	Large hooks	Blade length (μm)	Small hooks	Blade length (μm)	Total number of hooks
		Total length (μm)		Total length (μm)		
Host: sheep						
Thompson et al. 1984	UK	25.3 ± 0.9 (24.0–27.0)	12.4 ± 0.5 (12.0–13.0)	21.4 ± 1.5 (18.0–23.0)	8.6 ± 0.9 (7.0–10.0)	33.0 ± 1.8 (29.0–36.0)
Kumaratilake et al. 1986	UK	24.6 ± 0.9 (24.0–27.0)	12.8 ± 0.4 (12.0–13.0)	22.1 ± 0.8 (21.0–24.0)	9.8 ± 0.4 (9.0–10.0)	–
Ponce Gordo and Cuesta Bandera 1997	Spain	(23.7–25.4)	(12.1–13.0)	(20.7–22.4)	(8.3–9.2)	(32–38)
Our data	Poland	25.00 ± 0.76 (24–27)	12.55 ± 0.35 (12–13)	20.8 ± 1.13 (18–22)	9.03 ± 0.74 (8.1–10)	31.00 ± 2.52 (27–37)
Host: pig						
Eckert et al. 1993	Poland	29.1 ± 1.3 (26.2–31.1)	13.1 ± 0.7 (11.8–14.5)	24.4 ± 1.3 (21.4–26.7)	9.2 ± 0.6 (8.4–10.3)	33 ± 3.5 (30–38)
Yemets 2003	Ukraine	29.2 ± 0.16	14.1 ± 0.18	24.4 ± 0.1	11.1 ± 0.15	32.3
Ponce Gordo and Cuesta Bandera 1997	Spain	(25.0–27.4)	(13.1–13.6)	(21.1–22.7)	(9.1–9.7)	(32–37)
Host: human						
Cielecka et al. 2005	Poland	27.2 (24–32)	13.8 (10.8–17.6)	22.0 (14.4–26.0)	9.6 (7.2–13)	31.5 (28–39)
Cielecka et al. 2005	Ukraine	26.5 (24–28)	13.7 (12–15)	21.9 (21–25)	10.2 (9–11)	–
Ponce Gordo and Cuesta Bandera 1997	Spain	(21.9–23.0)	(12.0–12.8)	(19.3–20.3)	(8.7–9.4)	(38–52)

Data represent mean values ± SD. Ranges are given in parentheses

## Discussion

The presented data provide the first evidence of the presence of autochthonous infection with *E. granulosus* s. s. in Poland, which was detected in a sheep metacestode. We classified the sheep isolate from Podhale district located in the southernmost region of Poland as belonging to *E. granulosus* s. s. G1 based on sequences of mitochondrial genes. Apart from that, three human isolates and two pig isolates derived from central Poland were identified as *E. canadensis* G7.

The finding of endemic *E. granulosus* s. s. infection in Poland is of epidemiological significance given that its cysts are often fertile in humans, and numerous findings indicate their increased infectivity (or pathogenicity) compared to other *Echinococcus* species (Romig et al. 2015). According to the European Union summary report, 0.20% of pigs were infected with *E. granulosus* s. l. (44,981/21,973,398) and 0.49% prevalence rate (193/39,220) in sheep and goats was measured in 2015 during meat inspections at slaughterhouses (EFSA and ECDC 2016). Poland was ranked among the five EU countries exhibiting the highest number of animals infected with *E. granulosus* s. l. in this report. Although the pig is a major intermediate host mediating transmission of *E. granulosus* in the country given the high overall number of these infected animals, the role of sheep as an effective intermediate host for *E. granulosus* s. s. should also be taken into account, considering the current G1 finding. For humans, in 2011–2015, among 181 recorded cases of human echinococcosis at least 53 were caused by *E. granulosus* s. l. in Poland (Gołąb et al. 2016).

In *E. granulosus* s. l. isolates from Poland subjected to genotyping, the G7 genotype of *E. canadensis* was initially documented in 38 pigs and five humans (Kędra et al. 1999). G7 genotype was also found in a European beaver originating from north-eastern Poland (Tkach et al. 2002). In surveys targeted to human CE infections, Pawłowski and Stefaniak (2003) reported 16 patients infected with G7 from the Poznan region in central-western Poland, as was also reported by Dybicz et al. (2013) for 30 patients from central Poland. This was followed by another report of Dybicz et al. (2015), documenting seven cases of G7-infected patients and two cases of G1–infected patients coming from central Poland. Nevertheless, the authors stated that patients may have been infected with *E. granulosus* s. s. G1 outside Poland (Kazakhstan and Turkey, respectively), and thus they cannot be unambiguously regarded as indigenous. Hydatid cysts recently isolated from a patient in south-eastern Poland had homologous sequences to *E. canadensis* G7 (Šnábel et al. 2016). Overall, a total of 62 autochthonous G7 human infections have thus been until now documented in Poland including this study, along with 40 G7 pig infections, one G7 infection in the European beaver and one G1 infection in sheep presently recorded. According to our knowledge, *E. granulosus* from sheep were not genotyped to date in this country.

In central and northern Europe, *E. granulosus* s. s. (G1–G3 complex) is being detected only sporadically. Human autochthonous *E. granulosus* s. s. cases were recorded in Austria in two patients (Schneider et al. 2010) and in Hungary in one patient (Šnábel et al. 2016). In the latter human case originated from Békes county, the microvariant G1A identical to that seen in the present study was detected. Northward from Poland, *E. granulosus* s. s. G1 was recently identified in 4 (2.2%) urban dogs in Estonia (Tartu city) in the Baltic region, although *E. granulosus* tapeworms are primarily transmitted in the country through sylvatic cycle, maintained by moose and wolves harbouring *E. canadensis* G8 and G10 genotypes (Laurimaa et al. 2015). In a part of Russia belonging to eastern Europe, three *E. granulosus* s. s. G1 cases were documented in a domestic cat from Saint Petersburg (Konyaev et al. 2012b), in a sheep from Permskiy Krai and in a human from the Republic of Bashkiria (Konyaev et al. 2013). Sheep farming strongly affects the distribution of *E. granulosus* s. s in Europe, although involvement of cattle and goats as intermediate hosts may also be considerable in some regions.

The G1A microvariant, which bears the substitution C/T at position 56 relative to the common G1 type, commonly occurs in the southern Palearctic; we have found 206 records in GenBank entries and published articles with this sequence pattern worldwide (summarized list is in Table 1). According to the available data, the highest frequencies of this cosmopolitan G1A form were to date recorded in Asia and Africa, which account for 6.34% (136/2143) and 9.03% (47/436) of the total numbers of *E. granulosus* s. s.–genetically determined isolates in these continents. Interestingly, the frequency of G1A haplotype in Europe was 1.19% (14/1172) in compiled data that is a markedly lower distribution rate than those estimated in Asia and Africa. In main intermediate hosts of *E. granulosus* s. s., sheep and cattle, the proportion of rarer haplotypes in European populations has decreased with the increased distance from the domestication centre in the Middle East (Rannamäe et al. 2016). A similar scenario has likely occurred in their *Echinococcus* parasites, in which a part of genetic diversity was lost during their past distribution along the Mediterranean shore with livestock hosts. A low occurrence of G1A in Europe would also partially explain the lack of this haplotype in South America where only two findings (accounting for 0.20% frequency, 2/997) were to date documented in sheep from Brazil and in cattle from Argentina (Beato et al. 2013; Laurimae et al. 2016). The vast majority of cattle and sheep was imported to South America since sixteenth century from Europe (Arelovich et al. 2011), where the G1A haplotype is not abundant. Also several imports of livestock from Australia performed since the beginning of the twentieth century (Haag et al. 2004) could not contribute to G1A dispersal in South America considering its absence in the former continent according to available data.

There is an apparent link of the G1A haplotype with a cluster affiliated to the Turkish haplotype Tur35, which was detected as one of the two central haplotypes in a recent paleogeographical study of G1 distribution in the Mediterranean region, conducted by screening 8274 bp of mtDNA (Kinkar et al. 2016). Sixteen of 18 haplotypes from Turkey, Albania and Romania identified in the above study as derived from Tur35 isolate, located in eastern Turkey in the vicinity of a domestication centre for the majority of livestock species, possessed the 36C/T nucleotide exchange. Dominance in a frequency of G1A findings in Africa over Europe might reflect earlier arrival and establishment of *E. granulosus* with sheep and other livestock in North Africa than in Europe, but it is more likely caused due to stochastic bottleneck events associated with founder effects.

The G1A variant was so far identified in 76 sheep, 50 cattle, 40 humans, 8 water buffaloes, 7 donkeys, 5 camels, 3 goats, 1 wild boar, 1 mule and 1 red-tailed guenon within intermediate hosts (Table 1). Humans are globally infected with G1A in similar proportions as major livestock intermediate hosts (sheep to humans ratio 1.9, cattle to humans ratio 1.25), compared to the overall figure derived from published G1 records, encompassing 1478 sheep, 1492 cattle and 929 human isolates (ratio sheep to humans 1.59, cattle to humans ratio 1.61). Although a higher number of human G1A isolates was detected especially in comparison to cattle, differences in distributions of G1 and G1A genotypes in respective hosts are not yet statistically significant at *p* < 0.05 (Fisher’s exact test; *p* = 0.27 for cattle/human comparisons, *p* = 0.43 for sheep/human comparisons). Nucleotide substitutions seen in G1A genotype thus do not seemingly confer a higher virulence for this variant towards humans and do not present any epidemiological relevance.

Results obtained from rostellar hook morphology of protoscoleces from sheep of Polish origin corroborated our genetic determination in measuring shorter hooks than those from pig cysts from Poland and Ukraine (referenced data obtained from Eckert et al. 1997; Yemets 2003). The hooks were also shorter than those from humans in Poland and Ukraine (referenced by Cielecka et al. 2005) that had been later genetically confirmed as belonging to the “pig strain” (attributable to *E. canadensis* G7). Size differences were not so striking compared to pig isolates of Spanish origin (provided by Ponce Gordo and Cuesta Bandera 1997) in some hook characteristics; however, Spanish isolates could contain a mixture of G7 and G1 genotypes considering later reports on pig findings in the country (González et al. 2002; Daniel Mwambete et al. 2004).

The hooks had similar size to those originating from Spain and UK in the material obtained from sheep (Spain, UK) and humans (Spain) (referenced by Thompson et al. 1984; Kumaratilake et al. 1986; Ponce Gordo and Cuesta Bandera 1997). The cysts isolated from Spanish patients contained “sheep-cattle strain” (sensu Ponce Gordo and Cuesta Bandera 1997) that is now presumed to be *E. granulosus* s. s. given the territory and host concerned, which applies also for morphologically examined sheep samples from the UK by the above-mentioned authors. Nevertheless, data of rostellar hook morphology has to be interpreted with some caution given the effect of environmental factors, particularly host species (Hobbs et al. 1990).

High endemicity of human CE is being reported from areas with frequent transmission of *E. granulosus* s. s. The finding of infectious *E. granulosus* s. s. G1 in Poland, thus poses a threat to public health and may be relevant to the implementation of hydatid control in the country. As dosing regimes of dogs in control programmes are locally designed for the shorter development time of *E. canadensis* G7 (approx. 34 days p. i.), further measures should take into consideration simultaneous occurrence of more slowly developing *Echinococcus* s. s. with average maturation rate 45 days p. i. (Kumaratilake et al. 1983; Eckert et al. 1993). In the epidemiological situation in Poland characterized by intense transmission of *E. canadensis* G7 in domestic animals, a sporadic occurrence of *E. granulosus* s. s. should also be taken in account. Further metacestode samples should be analyzed from a variety of intermediate hosts (with special attention paid to sheep and humans) in concerned regions to provide a more detailed picture about the genotypic diversity of *E. granulosus* in Poland.
